# The complete mitochondrial genome of *Crocidura rapax* Allen, 1923 and its phylogenetic analyses

**DOI:** 10.1080/23802359.2025.2475839

**Published:** 2025-03-12

**Authors:** Zhu Liu, Lu Miao, Qiu-Ying Guo, Mei-Feng Han

**Affiliations:** College of Life Science and Technology, Mudanjiang Normal University, Mudanjiang, PR China

**Keywords:** *Crocidura rapax*, mitogenome, phylogenetic trees

## Abstract

This study aimed to examine the complete mitogenome sequence of *Crocidura rapax* Allen, 1923 using polymerase chain reaction. The mitochondrial genome of *C. rapax* is a circular double-stranded structure with a complete length of 17,517 bp. The mitochondrial genome of *C. rapax* included 13 protein-coding genes, one control region, 22 tRNA genes, two rRNA genes, and one origin of L-strand replication. This study confirmed the phylogenetic position of *C. rapax* in the *Crocidura* genus at the molecular level. The mitochondrial genome is of significant importance for elucidating the genetic background of *C. rapax.*

## Introduction

*Crocidura rapax* Allen, 1923 belongs to the order Eulipotyphla, family Soricidae, genus *Crocidura* (Ellerman and Morrison-Scott 1951)*. Crocidura* Wagler 1832 is the most speciose mammalian genus, comprising 198 species, about 45% of the species in the family Soricidae. It is found mainly in tropical and subtropical regions of Asia, Europe, and Africa, with a few species extending into the temperate zone (Burgin and He 2018). *C. rapax* was previously considered as a subspecies of *C. russula* (Ellerman and Morrison-Scott 1951). Later, it was elevated to a species according to molecular and morphological analyses (Jiang and Hoffmann 2001). The taxonomy and phylogeny of the *Crocidura* genus have been controversial. In this study, we report the mitochondrial genome of *C. rapax* under the correct species name (corrections to previously published articles; Chen, Chen, et al. 2016). The complete mitochondrial genome of *C. rapax* was sequenced, and the phylogenetic relationships within the *Crocidura* genus were analyzed.

## Materials and methods

**Figure 1.**
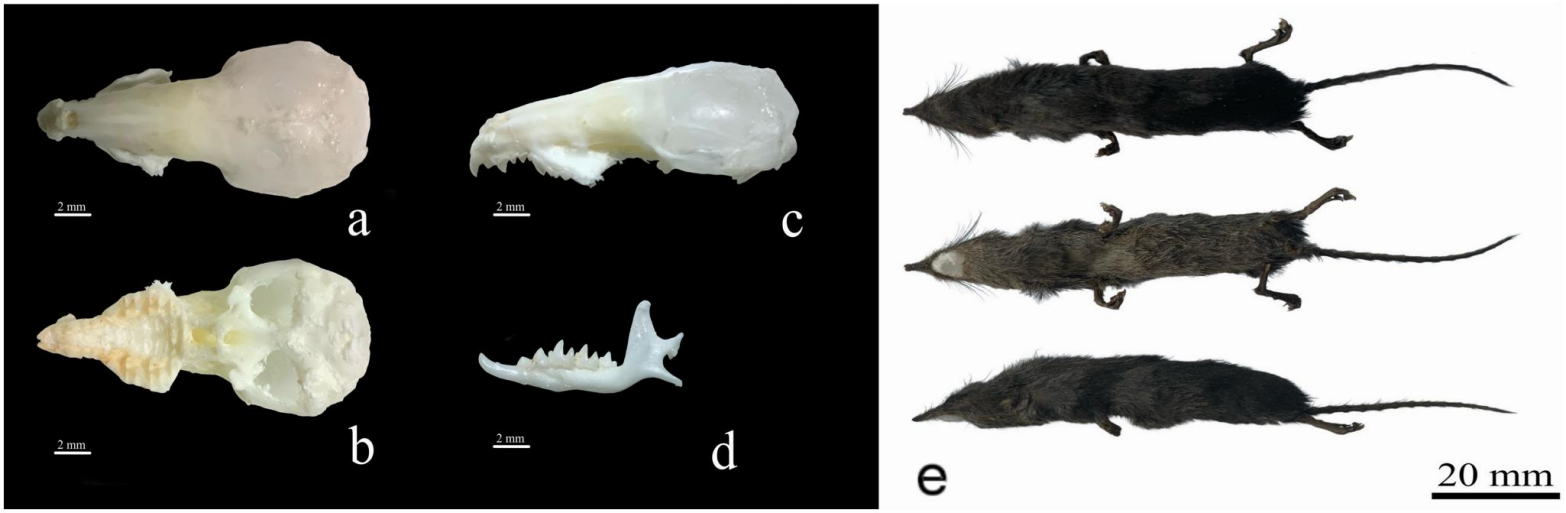
Pictures of external and skull morphologies (courtesy: Liu Zhu).

A *C. rapax* sample was collected from Liupanshui City (26°33′1″N, 104°57′18″E), Guizhou Province, China, in August 2023 (Figure 1). It was sampled in a dead state. *C. rapax* is not a protected animal in China. The Cyt b gene sequence of the specimen was blasted against the GenBank database. The sequence MW682360 was the most similar to the specimen from *C. rapax* (Song et al. 2021). The sample was stored at −75 °C before use and deposited at the Animal and Plant Herbarium of Mudanjiang Normal University (Liu Zhu, swxlz0@126.com) under the voucher number 2023LPS1. Genomic DNA was extracted from leg muscle of dead samples using the EasyPure genomic DNA kit (TransGen Biotech Co., Beijing, China). We designed 19 pairs of primers for polymerase chain reaction (PCR) based on the reported mitochondrial genome of *Crocidura* (Figures S1 and S2). The first-generation sequencing technology was used for sequencing in this study (ABI 3730 sequencer; Boshi Biotechnology Co. Ltd., Harbin, China). The sequences were assembled using DNAstar, analyzed, and adjusted manually. The annotation of the *C. rapax* mitochondrial genome was performed using web-based services MITOS (https://usegalaxy.eu) and software PhyloSuite v 1.2.2 (Zhang et al. 2020). The circular mitochondrial genome map of *C. rapax* was drawn using OGDRAW 1.3.1 (Greiner et al. 2019). In this study, the molecular phylogeny of *C. rapax* was investigated using the complete mitochondrial genomes of 12 species (*Crocidura anhuiensis*, *Crocidura attenuata*, *Crocidura dongyangjiangensis*, *Crocidura fuliginosa*, *Crocidura lasiura*, *Crocidura leucodon*, *Crocidura rapax*, *Crocidura shantungensis*, *Crocidura sibirica*, *Crocidura suaveolens*, *Crocidura tanakae*, and *Crocidura wuchihensis*) in the *Crocidura* genus deposited in the GenBank. The phylogenetic tree was constructed using 13 protein-coding genes of the complete mitochondrial genome through MEGA 11.0 software (Tamura et al. 2021). The phylogenetic tree was constructed using the Kimura 2-parameter model of maximum-likelihood method with 1000 bootstrap replications.

**Figure 2.**
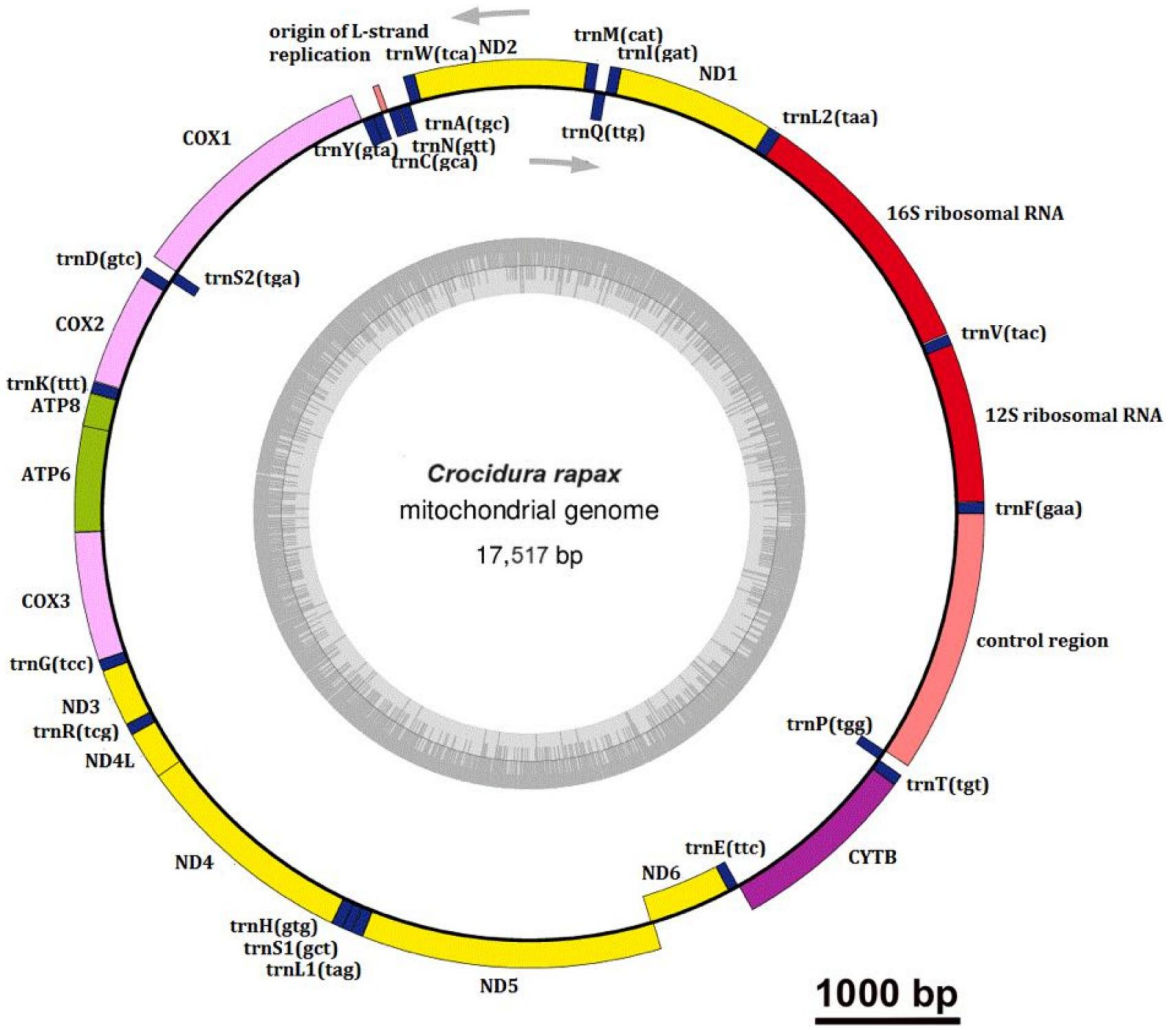
Circular mitochondrial genome map of *C. rapax.* The circle inside is the L strand, and the circle outside is H strand. Yellow, NADH gene; pink, COX gene; green, ATP gene; purple, other genes; blue, tRNA; red, rRNA; nude, origin of L-strand replication and control region.

## Results

**Figure 3.**
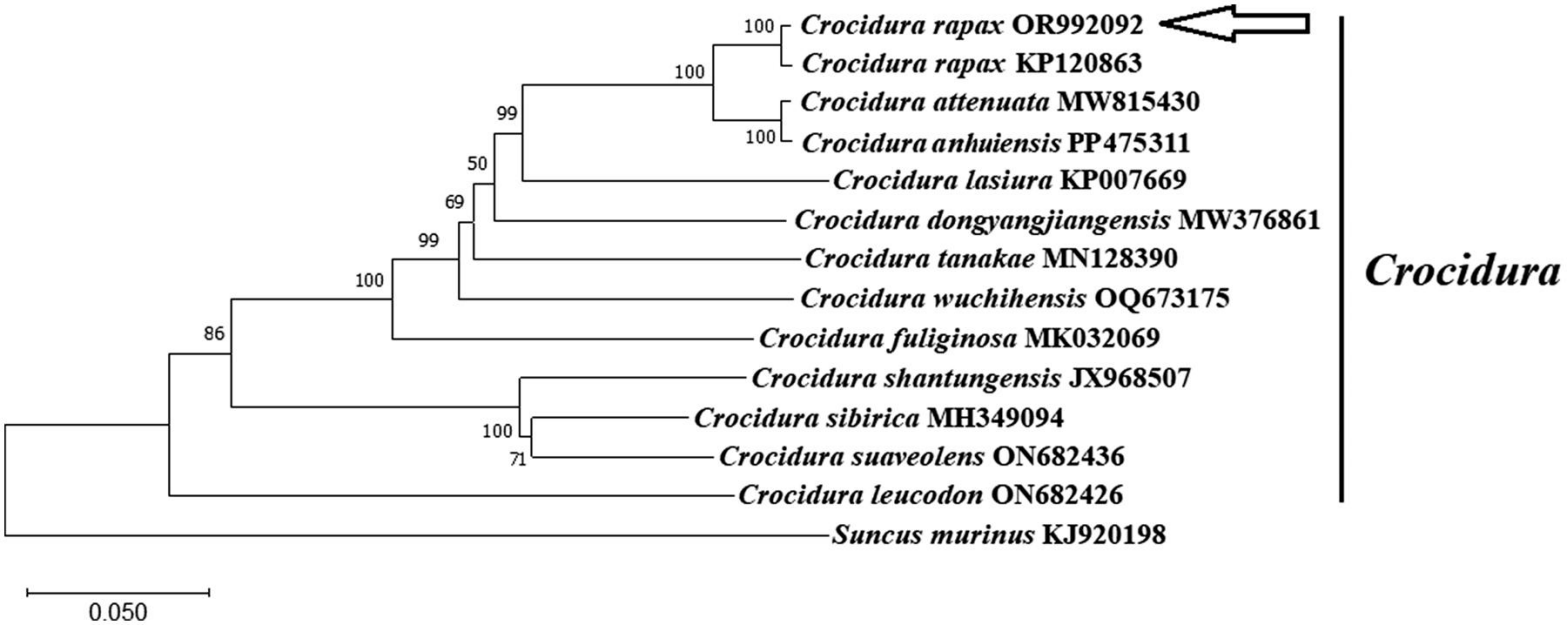
Phylogenetic tree was constructed using 13 protein-coding genes of the complete mitochondrial genome through MEGA 11.0 software, and also constructed using the Kimura 2-parameter model of maximum-likelihood method with 1000 bootstrap replications. *Crocidura rapax* (OR992092) is annotated and uploaded by this study. The following sequences were used: KP120863 (Chen, Chen, et al. 2016), MW815430 (Hinckley et al. 2022), PP475311 (unpublished), MW376861 (Li et al. 2021), MK032069 (Li et al. 2019), KR007669 (Kim et al. 2017), ON682426 (unpublished), JX968507 (Kim et al. 2013), MH349094 (Jiang et al. 2019), ON682436 (unpublished), MN128390 (Jin et al. 2019), and OQ673175 (Tu et al. 2023). The outgroup was KJ920198 (Chen, Wei, et al. 2016).

A circular double-stranded structure made up the mitochondrial genome of *C. rapax* (Figure 2). The length of the complete mitochondrial genome was 17,517 bp. The mitochondrial genome of *C. rapax* included 13 protein-coding genes, one control region, 22 tRNA genes, two rRNA genes, and one origin of L-strand replication (Figure 2). The total base composition of *C. rapax* mitochondrial genome was A (32.8%), T (31.9%), G (12.9%), and C (22.4%). We found significant A–T skew in base composition, especially in control regions and protein-coding genes. The *ND6* gene and eight tRNA genes of *C. rapax* were encoded on the L strand. The other mitochondrial genes were encoded on the H strand (Figure 2). GenBank received the annotated mitochondrial genome sequences with accession number OR992092. The control region of the mitochondrial genome existed between the tRNA-Pro and tRNA-Phe (Figure 2). The control region had no structural genes but had only promoters and regulatory sequences for replication and transcription. The total length of 13 protein-coding gene sequences was 11,411 bp. The lengths of 22 tRNA genes were between 59 and 75 bp. The length of L-strand replication origin (*OL*) was 41 bp. The Cyt b gene sequences of *C. attenuata* (KP120863) and *C. rapax* (OR992092) were compared, up to 99.47% similar, and more distantly related to the cyt b sequences of *C. attenuata* as reported in several previous studies. This means that KP120863 is in fact a species identification error for *C. rapax*, but not *C. attenuata*. The phylogenetic tree (Figure 3) showed that the eight species (*C. anhuiensis, C. attenuata*, *C. dongyangjiangensis*, *C. fuliginosa*, *C. lasiura*, *C. rapax*, *C. tanakae*, and *C. wuchihensis*) and *C. leucodon* in the *Crocidura* genus each formed independent branches. *C. shantungensis*, *C. sibirica*, and *C. suaveolens* formed independent branches (Figure 3). *C. rapax* was supported by the bootstrap values of 100% (Figure 3). Our results indicated that *C. anhuiensis*, *C. attenuata*, and *C. rapax* had a closer phylogenetic relationship (Figure 3).

## Discussion and conclusions

The arrangement of genes in *C. rapax* mitochondrial genome was consistent with that in other *Crocidura* genus species (Kim et al. 2013; Chen, Chen, et al. 2016; Kim et al. 2017; Jiang et al. 2019; Jin et al. 2019; Li et al. 2019, 2021; Tu et al. 2023). The Cyt b gene sequence is highly similar between *C. attenuata* KP120863 and *C. rapax* (OR992092), which is significantly different from the cyt b sequences of *C. attenuata* reported in several previous studies. This evidence supports the correct classification of KP120863 as *C. rapax*. For the first time, this study reports the mitochondrial genome of *C. rapax* under the correct species name, providing a widely recognized reference standard for this species. Moreover, it validates the phylogenetic position of *C. rapax* within the *Crocidura* genus at the molecular level, which is of significant importance to the genetic background of *C. rapax.*

## Supplementary Material

PCR Primers.doc

## Data Availability

The data supporting the findings of this study are publicly accessible on the GenBank website at https://www.ncbi.nlm.nih.gov/, under the reference number OR992092. The additional data can be accessed at BioSample: SAMN40990995, SRA: SRR29203679, and BioProject: PRJNA1101227. Raw data can be downloaded at https://www.ncbi.nlm.nih.gov/sra/?term=SRR29203679.
