# Supplementary material for: The complete mitochondrial genome of Crocidura rapax Allen, 1923 and its phylogenetic analyses
Source: Mitochondrial DNA B Resour. 2025 Mar 12;10(4):288–91. doi: 10.1080/23802359.2025.2475839 (PMC11905319; doi:10.1080/23802359.2025.2475839)
Supplement: PCR Primers.doc [file TMDN_A_2475839_SM8633.doc]

| Primer | Sequence(5'-3') |
| --- | --- |
| HNZSQ1F-1  HNZSQ1F-2 | CTGAAAATGCTTAGATGGGTAT  CTTCATGGCCTTATTCAGTTAA |
| HNZSQ1B-1  HNZSQ1B-2 | AGGCATAATAGTAAGCCAAAGT  TCATGAATAGCTCGTCTGGTT |
| TM2-1  TM2-2 | TACCGCAAGGGAAAGATGAAA  TAGGGTAACTTGGTCCGTTGA |
| HNSQTM3-1  HNSQTM3-2 | GAGAAGACCCTATGGAGCTTA  AGGGTATGAAGCACGTACTCA |
| HNSQTM4-1  HNSQTM4-2 | CCGCAGGCCCATTCGCATTAT  TAAATCCTGTTAGTGGGGGAAG |
| HNZSQ5F-1  HNZSQ5F -2 | CCTATCTCTCACCTGAAACAA  GCAAATTCAAAGGAGCAGCTT |
| HNSQTM6-1  HNSQTM6-2 | CTTGTTCGTCTGATCCGTCTT  GTTTCCACTGCTTGAGCATCT |
| HNSQTM7-1  HNSQTM7-2 | AGGCTTACAAGATGCCACATC  AAATGGGCTAGTGATGCCTTG |
| HNSQTM8-1  HNSQTM8-2 | GGCCAAACATGAACACTTATAC  TGGAGCCATAAATTCCATCTGA |
| HNSQTM9-1  HNSQTM9-2 | TACGTCCGTCCTATTAGCCTC  CCCTCTAAGCATAGTAGAGATG |
| TWTM10-1  TWTM10-2 | TCATAATTATCAAATGTCTCTAGT  ATAAGAGATTTAAGRTCTGTTTG |
| TWTM11-1  TWTM11-2 | GCTTCACTTGTGGCTACCAA  CTTTTATTTGGAGTTGCACCAA |
| TWTM12-1  TWTM12-2 | GCAAGAACTGCTAATTCATGC  GCGTTGGTATACGACGTGTT |
| TWTM13-1  TWTM13-2 | GGTAGCYTAGCATTAACAGG  TGGTGTCTGATGTATAGTGTAT |
| HNZSQ14-1N  HNZSQ14-2N  HNZSQ15F-1  HNZSQ15F-2  HNZSQ15B-1  HNZSQ15B-2 | GGTCAATTAGCCTCCGTAATT  GTACATGCTTATATGCATGGG  CCGTGCATATTAGCATGTACT  ACCAGATGCCAGGTAAAGTGT  ACCAACTCACGTGAAACCAAC  ACCAGATAATAAGAAGGCTGG |
